# 

**Published:** 1890-05

**Authors:** 


### BOOKS AND PAMPHLETS RECEIVED.

Leçons de Gynécologié Opératoire par Vulliet, Profésseur à la Faculté de Médicine de Genéve, etc., et Lutaud, Professeur libre de Gynécologie à l'ecole practique, etc. Deuxiéme édition entirément refondue, avec 200 figures intercalées dans le texte. 8vo, paper, pp. 500. Paris: A. Maloine, 91. Boulevard Sant-Germain. 1890.

May's Diseases of Women. Being a Concise and Systematic Exposition of the Theory and Practice of Gynecology. For the use of Students and Practitioners. Second edition, revised by Leonard S. Rau, M. D., attending physician to Harlem Hospital, out-patient department, New York, etc., with thirty-one illustrations on wood. Small 8vo, pp. 373. Philadelphia: Lea Brothers & Co. 1890.

Transactions of the Eleventh Annual Meeting of the American Laryngological Association, for the year 1889. 8vo, cloth; pp. 150. New York: D. Appleton & Co. 1890.

Wood's Medical and Surgical Monographs, Vol. vi., No. 1. April, 1890. Contents: The Human Foot; its Form and Structure, Functions and Clothing. By Thomas S. Ellis, M. R. C. S. Modern Cremation; its History and Practice. By Sir H. Thompson, F. R. C. S. Aphasia, a contribution to the subject of the Dissolution of Speech from Cerebral Disease. New York: William Wood & Co. 1890.

Transactions of the Medical Society of the State of North Carolina. Thirty-sixth Annual Session. 1889. Wilmington, N. C. Jackson & Bell. 1890.

Health Notes for Students. By Burt G. Wilder, B. S., M. D., Professor of Physiology, Comparative Anatomy, and Zoölogy in Cornell University. Second edition revised and enlarged. New York and London: G. P. Putnam's Sons. 1890.

An Experimental Study of Intestinal Anastomosis. By John D. S. Davis, M. D., Birmingham, Ala. Reprint from the *Times and Register*, January 25, 1890.

Epicystic Surgical Fistula for the Relief of Vesical Catarrh. By John D. S. Davis, M. D., Birmingham, Ala. Reprint from *Journal American Medical Association*, February 8, 1890.

Cholecystotomy. Report of a Case with Illustrations. By Edwin Ricketts, M. D., Cincinnati, O. Reprint from the *Lancet-Clinic*, December 21, 1889.

Some Operations on the Gall Bladder. With a report of five cases. By Edwin Ricketts, Cincinnati, O. Reprint *Lancet-Clinic*, 1889.

A Rational Method of Obtaining Extension of the Spinal Cord and Column. By Charles F. Stillman, M. S., M. D., Chicago. With a discussion. Reprint from Transactions of the Chicago Medical Society.

The Identification of Criminals. Its Value as a Prevention of Crime, etc. By Charles E. Felton, Superintendent of the House of Correction, Chicago, Ill. Reprint. Read at the Congress of the National Prison Association, at Nashville, Tenn., November, 1889.

Sudden Emptying of the Bladder, as a Cause of Cystorrhagia. By Walter P. Manton, M. D , Detroit, Mich. Reprint from the *American Lancet*, 1890.

Modern Methods of Local Treatment in Skin Diseases by Several Authors. New York: Johnson & Johnson. 1890.

Hemoglobin Compound, or Bullock's Blood in Therapeutics. By F. E. Stewart, M. D., Ph. G. Detroit and New York: Parke Davis & Co. 1890.

The Early Detection of Pulmonary Consumption. By Wm. B. Canfield, A. M., M. D. Baltimore, Md. Reprint *Maryland Medical Journal*. 1890.

Report of the Section on Practice of Medicine. By Wm. B. Canfield, A. M., M. D. Baltimore, Md. Reprint Transactions Medical and Chirurgical Faculty of the State of Maryland. 1889.

Some Complications of Chronic Endarteritis. By Wm. B. Canfield, A. M., M.D., Baltimore, Md. Reprint from the *Medical Record*, October 8, 1887.

Mr. Bellamy and the New Nationalist Party. By Francis A. Walker, President of the Massachusetts Institute of Technology. Reprint from the *Atlantic Monthly* for February, 1890.

Tobacco Amblyopia. By Leartus Connor, A. M., M. D., Detroit, Michigan. Reprint from *Journal of American Medical Association*, February 15, 1889.

The Use of Menthol in Diseases of the Upper Air-Passages. By Frank Hamilton Potter, M. D., Buffalo, N. Y. Reprint from *Journal of American Medical Association*, February 1, 1890.

The *Lactopeptin Medical Annual* for 1890. Published by the New York Pharmacal Association.

Remarks of Gen. N. M. Curtis in Support of his Bill, No. 356, to Abolish Capital Punishment. In Assembly, March 26, 1890. From the Author.

Optic Neuritis and Its Significance as a Symptom. By Alvin A. Hubbell, M. D , Buffalo, N. Y. Reprint from the *Buffalo Medical and Surgical Journal*. February 1890.

An Experimental Study of Lesions arising from Severe Concussions. By B. A. Watson, A. M., M. D. P. Blakiston, Son & Co., Philadelphia, Printers, 1890.

Special Hospitals for the Treatment of Tuberculosis. By Lawrence F. Flick, M. D. Reprint from *The Times and Register*. March 15, 1890.

The Value of Buffalo Lithia Water in the Treatment of Lithiasis and Kindred Diseases of the Kidney. By Wm. C. Wile, A. M., M. D. Reprint from *New England Medical Monthly* for February, 1890.

The Mineral Springs of Colorado. By Charles Denison, M. D., Denver, Colorado. Reprint from Transactions American Climatological Association. June, 1889.

The Extension-Windlass Method of Treating Fractures of Long Bones. By Charles Denison, A. M., M. D., Denver, Colorado. Report from the *Medical Record*, August 18, 1888.

The Four Commencements. Valedictory Addresses to the Graduates of the Medical Department, University of Louisville, February 28, 1890. By J. M. Bodine, M. D., Professor of Anatomy and Dean of the Faculty. Published by request.

Forty-seventh Annual Report of the Managers of the State Lunatic Asylum at Utica, N. Y., for 1889.

The History of Federal and State Aid to Higher Education in the United States. By Frank W. Blackmar, Ph. D., Bureau of Education, Circular of Information. No. 1. 1890. Washington: Government Printing Office. 1890.

Proceedings of the Department of Superintendence of the National Educational Association, at its meeting held in Washington, March 6, 8, 1889. Bureau of Education, Circular of Information. No. 2. 1890. Washington: Government Printing Office. 1890.

Annual Report of the Corporation Counsel of the City of Buffalo, for the year ending December 31, 1889. W. F. Worthington, Corporation Counsel. Times Printing House. 1890.

Annual Report of the New York Orthopedic Dispensary and Hospital, (for Children with Spine and Hip Diseases and other Deformities,) for the year ending September 30, 1889. Situated at 126 East Fifty-ninth street. Open from 1 to 3 P. M. daily, except Sundays and holidays. Printed by order of the Board of Trustees. 1890.

Inflammation of the Vermiform Appendix; its Results, Diagnosis, and Treatment. Together with reports of seven cases of excision of the vermiform appendix, for perforative appendicitis, with exhibition of five of the patients. By Thomas G. Morton, M. D., Surgeon to the Pennsylvania Hospital, etc. Read before the College of Physicians of Philadelphia, January 1, 1890. Philadelphia: J. B. Lippincott Company. 1890.

Anomalies of the Ocular Muscles. Third paper, by Dr. George T. Stevens, New York. Reprint from the Archives of Ophthalmology. Vol. xviii. No. 4. 1889.

Accidents, Complications, and Results, following Internal Urethrotomy upon 120 Cases of Stricture. By George E. Brewer, M. D., Clinical Assistant in Genito-Urinary Diseases, College of Physicians and Surgeons, New York. Reprint from the *International Journal of Surgery*, January, 1890.

A Failure in Brain Surgery. By Hal. C. Wyman, M. D., Detroit, Mich. Reprint from the *Medical News*, February, 1890.

Chloralamid Schering; a Hypnotic discovered by Dr. J. Von Schering, of Strassburg. Second revised edition. Published by Lehn & Fink, New York. 1890.

Sketch of the Late Dr. J. Edward Turner, the Founder of Inebriate Asylums. By T. D. Crothers, M. D., Hartford, Conn. Reprint from the *Quarterly Journal of Inebriety*, 1889.

Fifteenth Annual Report of the Central New York Institution for Deaf Mutes at Rome, N. Y., for the year ending September 30, 1889. Albany: James B. Lyon, State Printer. 1890.

Twentieth Annual Report of the Buffalo Park Commissioners. January, 1890. Buffalo: Haas & Klein. 1890.

The National University, Chicago. Announcement, 1889-90.

Yale University. Department of Medicine (Yale Medical School). Reprinted from the University Catalogue, 1890.

Prospectus of the London Post-Graduate Course, 1890.

University of Cincinnati. Programme of the Academic Department, 1890–91.

Medical and Surgical Society Transactions. Catalogue. W. J. Dornan, Philadelphia, Pa.

Abstract of Sanitary Reports. U. S. Marine Hospital Service. Vol v., Nos. 10, 11, 12, 13, 14, 15 and 16.

Monthly Bulletin of the New York State Board of Health for February, 1890.

Health in Michigan. February and March, 1890. Bulletins of State Board of Health.

Tennessee State Board of Health Bulletins, March 20 and April 20, 1890.

Report of Deaths and Contagious Diseases for February and March, 1890. Newport, R. I.

